# Mr. James Startin

**Published:** 1910-06-11

**Authors:** 


The death is announced of Mr. James Startin, aged 59 years. He became an M.R.C.S. in 1876, having studied at St. Thomas's. His principal appointment was that of senior surgeon and lecturer to the London Skin Hospital. He was also connected with many medical societies, but perhaps his chief energies were devoted to writing, he being the author of many lectures, written and spoken, and a frequent contributor to the medical papers. He also invented a hypodermic syringe and a toughened-gold needle for electrolysis.

